# Promoter of Vegetable Soybean *GmTIP1;6* Responds to Diverse Abiotic Stresses and Hormone Signals in Transgenic *Arabidopsis*

**DOI:** 10.3390/ijms232012684

**Published:** 2022-10-21

**Authors:** Zhijuan Feng, Na Liu, Guwen Zhang, Yuanpeng Bu, Bin Wang, Yaming Gong

**Affiliations:** Institute of Vegetables, State Key Laboratory for Managing Biotic and Chemical Threats to the Quality and Safety of Agro-Products, Key Laboratory of Vegetable Legumes Germplasm Enhancement and Molecular Breeding in Southern China of Ministry of Agriculture and Rural Affairs, Zhejiang Academy of Agricultural Sciences, Hangzhou 310021, China

**Keywords:** vegetable soybean, aquaporin, promoter, GUS, transgenic *Arabidopsis*, abiotic stresses, hormone signals, *GmTIP1;6*

## Abstract

Tonoplast intrinsic proteins (TIPs), a sub-family of aquaporins (AQPs), are known to play important roles in plant abiotic stress responses. However, evidence for the promoters of TIPs involvement in abiotic stress processes remains scarce. In this study, the promoter of the vegetable soybean *GmTIP1;6* gene, which had the highest similarity to TIP1-type AQPs from other plants, was cloned. Expression pattern analyses indicated that the *GmTIP1;6* gene was dramatically induced by drought, salt, abscisic acid (ABA), and methyl jasmonate (MeJA) stimuli. Promoter analyses revealed that the *GmTIP1;6* promoter contained drought, ABA, and MeJA *cis*-acting elements. Histochemical staining of the *GmTIP1;6* promoter in transgenic *Arabidopsis* corroborated that it was strongly expressed in the vascular bundles of leaves, stems, and roots. Beta-glucuronidase (GUS) activity assays showed that the activities of the *GmTIP1;6* promoter were enhanced by different concentrations of polyethylene glycol 6000 (PEG 6000), NaCl, ABA, and MEJA treatments. Integrating these results revealed that the *GmTIP1;6* promoter could be applied for improving the tolerance to abiotic stresses of the transgenic plants by promoting the expression of vegetable soybean *AQPs*.

## 1. Introduction

Vegetable soybean (*Glycine max* L.), also called “mao dou” in China and “edamame” in Japan, is a soybean harvested at the R6 growth stage when the seeds are still green and immature but fully filled inside the pods [1]. Vegetable soybean has huge nutritional value, and is rich in carbohydrate, protein, vitamin, mineral, isoflavone, fiber, and folate [2]. It serves as one of the most important legumes in many Asian countries, and has gained widespread acceptance in the USA and some European and African countries [3]. China is the largest producer, consumer, and exporter of vegetable soybean in the world [4,5]. Drought, salt, and cold stresses severely affected the yield and quality of vegetable soybeans [6,7,8]. Currently, researches on the abiotic stress response mechanism of vegetable soybean are extremely inadequate [9,10,11,12]. It is promising to discover genes responsive to environmental factors and develop cultivars tolerant to abiotic stresses for vegetable soybean, especially given the impacts of global climate changes.

Aquaporins (AQPs), known as membrane channel proteins, have imperative functions in modulating multiple plant abiotic stress tolerances [13]. Since the first discovery of AtTIP1;1 from *Arabidopsis* [14], a large number of AQPs have been identified in a wide range of plants species. According to the sequence similarity, AQPs could be classified into plasma membrane intrinsic proteins (PIPs), tonoplast intrinsic proteins (TIPs), NOD26-like intrinsic proteins (NIPs), small basic intrinsic proteins (SIPs), and the unrecognized X intrinsic proteins (XIPs) [15,16,17]. Among them, TIPs were generally localized in the tonoplast, mediating water and small neutral molecule exchange between the cytoplasm and vacuole. The vacuole played vital roles in space filling, osmotic adjustment, storage, and digestion [18]. Many TIPs have been investigated, including 24 GmTIPs in soybean, 11 AtTIPs in *Arabidopsis*, 10 OsTIPs in rice, and 12 ZmTIPs in maize [19,20,21,22]. Nevertheless, the functional explorations of most TIPs have not been attained yet. Different TIP members consisted of distinct trans-membrane (TM) domains, Asn-Pro-Ala (NPA) motifs, aromatic/arginine (ar/R) selectivity filters, and Froger’s positions (FPs), which determined the substrate specificities. Different *TIP* genes exhibited specific expression profiles under environmental stresses [23,24]. Manipulation of *TIP* gene expressions is the key in defining distinctive biological roles in plants.

Promoters regulate the expression pattern of particular genes at the transcriptional level [25,26,27,28]. Stress responsive promoters had considerable prospect for use in plant genetic engineering for improving stress tolerance [29]. Several studies have indicated that developmental cues, as well as environmental stress signals, regulated the function of *TIP* promoters. In bananas, the beta-glucuronidase (GUS) activity of the *MaTIP1;2* promoter was elevated under drought and salt stresses in transgenic *Arabidopsis* [30]. In soybeans, the GUS activity of the *GmTIP2;3* promoter was observed in the roots, stems, and leaves and preferentially expressed in the steles of roots and stems and down-regulated under polyethylene glycol 6000 (PEG 6000) and abscisic acid (ABA) stress conditions in transgenic lotus [31]. The *GmTIP2;6* promoter was strongly induced in hypocotyls, vascular bundles, and leaf trichomes after l-aminocyclopropane-l-carboxylic acid (ACC) and heat stress treatments in transgenic *Arabidopsis* [19]. However, the functions of most *TIP* promoters are still unknown. It is beneficial to explore the roles of other functionally unknown *TIP* promoters.

In this study, one novel promoter of *GmTIP1;6* was isolated and characterized from vegetable soybean. Histochemical patterns and transcriptional activities of the *GmTIP1;6* promoter under abiotic stresses were further examined in transgenic *Arabidopsis*. The results proved that the GUS activities of *GmTIP1;6* promoters were abundantly present in the vascular tissues of leaves, stems, and roots and remarkably enhanced by drought, salt, ABA, and methyl jasmonate (MeJA) stresses. These findings will contribute to clarify the transcriptional regulation mechanism of the *GmTIP1;6* gene and provide stress responsive promoter resource for plant genetic engineering.

## 2. Results

### 2.1. Phylogenetic Analysis of GmTIP1;6 Gene

**Figure 1:**
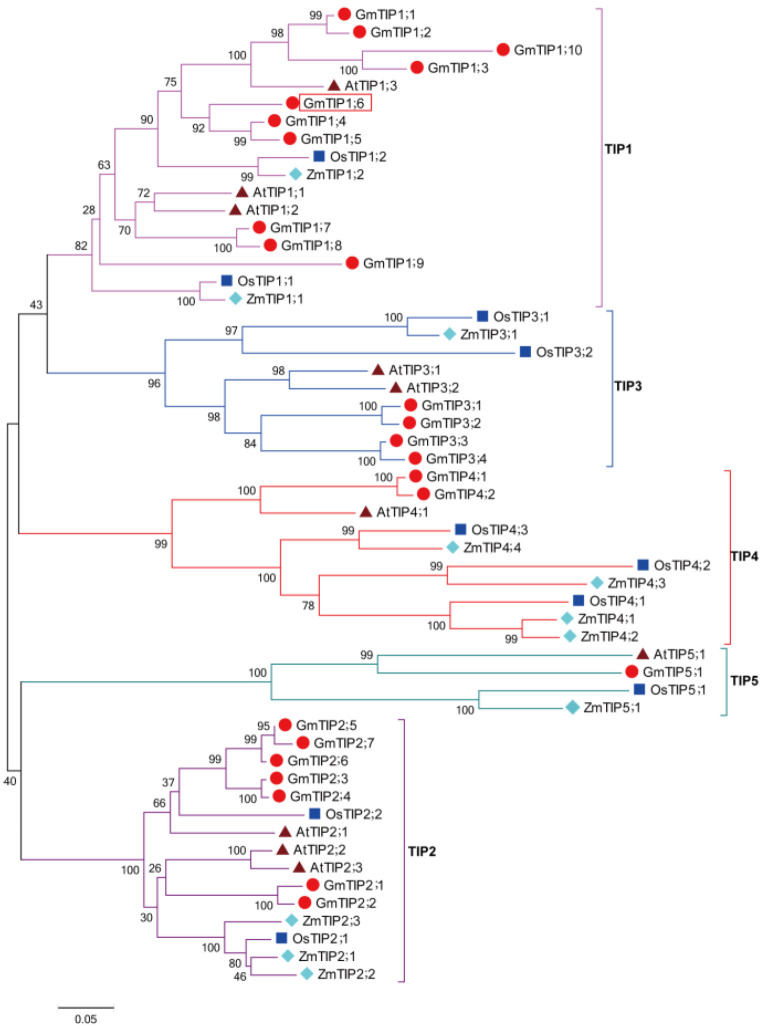
Phylogenetic relationship of GmTIP1;6 from soybean, *Arabidopsis*, rice and maize TIPs. TIPs were divided into five groups (TIP1, TIP2, TIP3, TIP4, and TIP5), which were marked with different colors. Soybean TIPs (GmTIPs), *Arabidopsis* TIPs (AtTIPs), rice TIPs (OsTIPs), and maize TIPs (ZmTIPs) were marked with red spots, purple triangles, dark blue squares, and light blue diamonds, respectively. GmTIP1;6 was marked with the red box.

To determine the evolutionary relationship and classification of GmTIP1;6, a phylogenetic tree was created using soybean TIPs (GmTIPs), *Arabidopsis* TIPs (AtTIPs), rice TIPs (OsTIPs), and maize TIPs (ZmTIPs). The result showed that all TIPs were categorized into five groups: TIP1, TIP2, TIP3, TIP4, and TIP5. GmTIP1;6 had the highest similarity to TIP1-type proteins from other plants and the best orthologic match of GmTIP1;6 was GmTIP1;5, AtTIP1;3, OsTIP1;2, and ZmTIP1;2 (Figure 1).

### 2.2. Expression Patterns of GmTIP1;6 Gene

**Figure 2:**
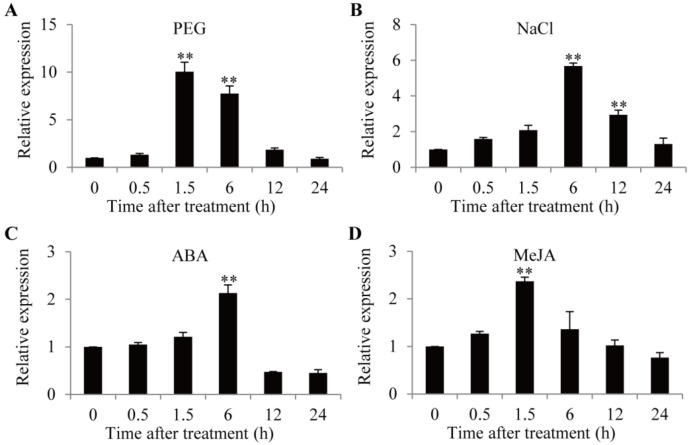
Expression patterns of *GmTIP1;6* under drought, salt, ABA, and MeJA stress treatments in vegetable soybean seedlings. Vegetable soybean seedlings were treated with 20% PEG6000 (**A**), 250 mM NaCl (**B**), 100 µM ABA (**C**), and 100 µM MeJA (**D**). ** indicated significant differences in comparison with the control treatment at *p <* 0.01 (*t*-test).

To confirm the roles of *GmTIP1;6* in response to abiotic stresses and hormone signals, the expression patterns of *GmTIP1;6* in vegetable soybean seedlings that were treated with PEG6000, NaCl, ABA, MeJA, GA3, or NAA were analyzed by qRT-PCR. After a 20% PEG6000 treatment, the expression of *GmTIP1;6* reached the most dramatic induction (10.1 fold) at 1.5 h (Figure 2A). After a 250 mM NaCl treatment, the expression of *GmTIP1;6* was significantly induced (5.7 fold) at 6.0 h (Figure 2B). When treated with a 100 µM ABA treatment, the expression of *GmTIP1;6* was up-regulated (2.1 fold) at 6.0 h (Figure 2C). After a 100 µM MeJA treatment, the expression of *GmTIP1;6* increased (2.4 fold) at 1.5 h (Figure 2D). When exposed to a 100 nM gibberellin 3 (GA3) or a 100 nM 1-naphthaleneacetic acid (NAA) treatment, the expression of *GmTIP1;6* presented no obvious change or decreased (Figure S1). The result indicated that *GmTIP1;6* responded to drought, salt, ABA, and MeJA stimuli.

### 2.3. Isolation and Cis-Acting Element Distribution of GmTIP1;6 Promoter

**Figure 3:**
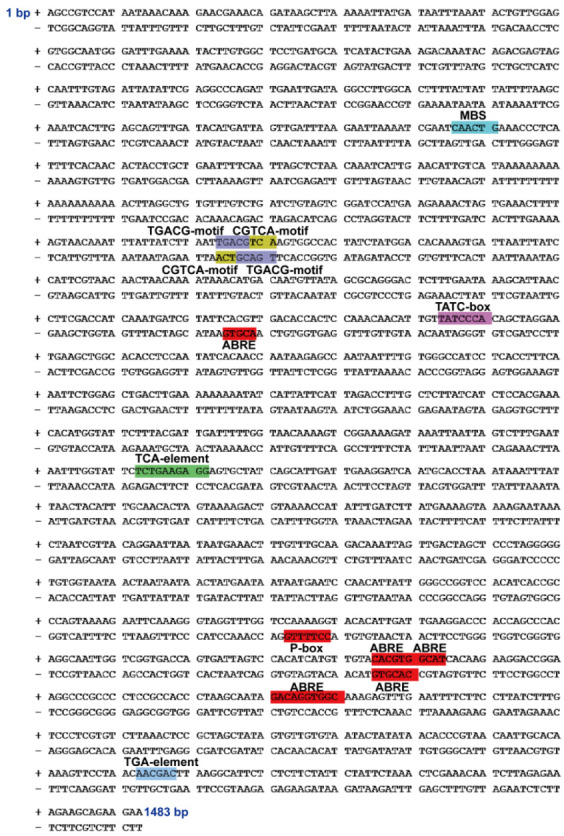
The 1.483 kb promoter sequence and *cis*-acting element distribution of the *GmTIP1;6*. + and − represented the sense and antisense strand, respectively. Different elements with different core sequences in the promoter were marked by different colors.

A 1.483 kb promoter sequence, upstream of the ATG start codon of the *GmTIP1;6* gene, was cloned. The *cis*-acting elements of *GmTIP1;6* promoter were identified by the PlantCARE database. Many abiotic stress and hormone signal-related elements were found, including one drought stress response element (MBS), five ABA response elements (ABRE), four MeJA response elements (CGTCA and TGACG motifs), two GA response elements (P and TATC boxes), and one auxin response element (TGA element) (Figure 3; Table 1). The result suggested that the *GmTIP1;6* promoter might be involved in diverse abiotic stress and hormone signal responses.

### 2.4. Histochemical Localization of GmTIP1;6 Promoter

**Figure 4:**
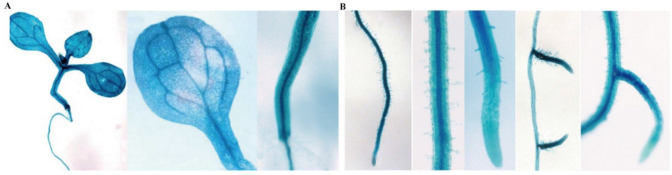
Tissue expression patterns of *GmTIP1;6* promoter in transgenic *Arabidopsis*. GUS staining for the aerial (**A**) and underground (**B**) parts of proGmTIP1;6-GUS transgenic *Arabidopsis* seedlings.

To evaluate the functions of the *GmTIP1;6* promoter, it was fused to the GUS reporter gene and transformed into *Arabidopsis*. GUS staining was observed in the transgenic seedlings, including leaves, stems, and roots. In the aerial part, GUS staining was strongly detected in the vascular bundles of leaves and stems (Figure 4A). In the underground part, GUS staining was obviously detected in the vascular bundles of primary roots and root hairs, except for the root apexes (Figure 4B). It was evident that *GmTIP1;6* promoter played a key role in the vascular tissues of seedlings.

### 2.5. Activities of GmTIP1;6 Promoter in Response to Drought and Salt Stresses

**Figure 5:**
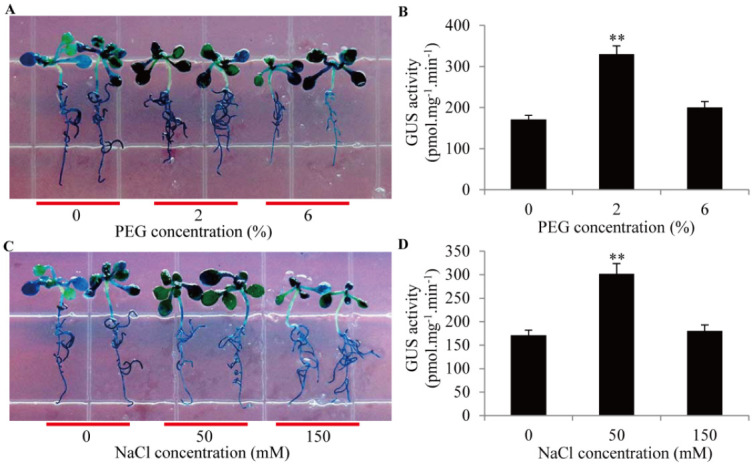
GUS activities of the *GmTIP1;6* promoter under drought and salt stress treatments in transgenic *Arabidopsis*. (**A**,**C**) GUS staining for the proGmTIP1;6-GUS transgenic seedlings treated with 2–6% PEG6000 or 50–150 mM NaCl. (**B**,**D**) Activity analyses of GUS protein in transgenic seedlings under 2–6% PEG6000 or 50–150 mM NaCl treatment. ** indicated significant differences in comparison with the control treatment, *p <* 0.01 (*t*-test).

To explore the roles of the *GmTIP1;6* promoter in response to drought and salt stresses, transgenic *Arabidopsis* seedlings were subjected to 2–6% PEG6000 or 50–150 mM NaCl, and GUS activities were compared. After PEG treatment, GUS staining was sharply enhanced (Figure 5A). Stronger GUS activity was detected under 2% PEG6000 than that under 6% PEG6000 (Figure 5B). Similarly, after NaCl treatment, GUS staining was remarkably induced (Figure 5C). GUS activity under 50 mM NaCl was higher than that under 150 mM NaCl (Figure 5D). The result confirmed that the activities of the *GmTIP1;6* promoter could be elevated by drought and salt stresses.

### 2.6. Activities of GmTIP1;6 Promoter in Response to ABA and MeJA Signals

**Figure 6:**
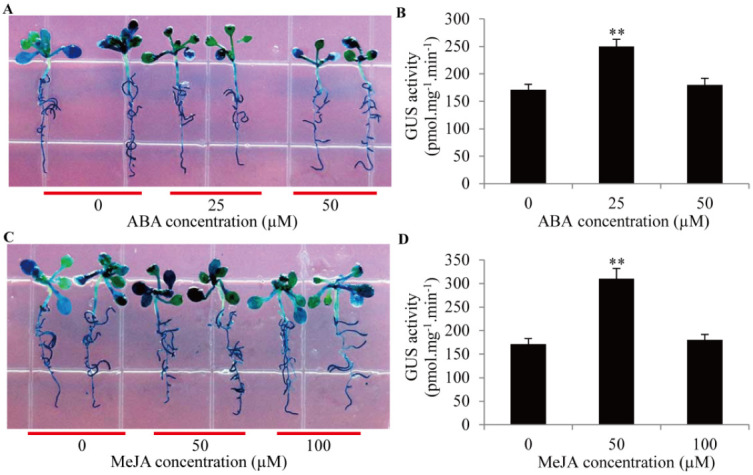
GUS activities of the *GmTIP1;6* promoter under ABA and MeJA hormone treatments in transgenic *Arabidopsis***.** (**A**,**C**) GUS staining for proGmTIP1;6-GUS transgenic seedlings treated with different hormone signals (25–50 µM ABA or 50–100 µM MeJA). (**B**,**D**) Activity analyses of GUS protein in transgenic seedlings under different hormone treatments. ** indicated significant differences in comparison with the control treatment, *p <* 0.01 (*t*-test).

To investigate the roles of the *GmTIP1;6* promoter in response to ABA and MeJA signals, transgenic seedlings were treated with 25–50 µM ABA or 50–100 µM MeJA, and GUS activities were contrasted. ABA and MeJA treatments highly promoted the GUS activity (Figure 6A,C). GUS activity after a 25 µM ABA treatment exhibited a greater increase than that after a 50 µM ABA treatment (Figure 6B). Similarly, GUS activity after a 50 µM MeJA treatment displayed more abundance than that after a 100 µM MeJA treatment (Figure 6D). The result verified that the activities of the *GmTIP1;6* promoter could be enhanced by ABA and MeJA signals.

## 3. Discussion

Harsh environmental conditions negatively influenced the vegetable soybean production [32,33,34,35,36]. In recent years, due to high nutritional benefit, the cultivation area of vegetable soybean had gradually expanded worldwide. Simultaneously, the risks of vegetable soybean facing various abiotic stresses significantly increased owing to global climate changes. However, candidate genes available for improving vegetable soybean abiotic stress tolerance are still scarce.

AQPs extensively participated in plant adaptation to variable environmental stresses. TIPs, one sub-family of AQPs, supported the function of multifaceted vacuoles by facilitating the transport of water and other small solutes [37,38,39]. Regarding the sequence homology, TIPs could be further categorized into five groups: TIP1, TIP2, TIP3, TIP4, and TIP5 [40]. TIP homologs were localized in distinct vacuolar subtypes based on the specific functions [41,42,43,44]. Gene functions of different TIP members showed complex patterns with no generality. For instance, overexpressing of tomato *SlTIP2;2*, cotton *GhTIP1;1*, and *GhTIP2;1*, *Thellungiella salsuginea TsTIP1;2*, *Jatropha curcas* *JcTIP1;3*, *Passiflora edulis PeTIP3;2* and wheat *TaTIP4;1*, increased drought, salt, cold, osmotic, and oxidative stress resistances by regulating the water relation, ROS balance, the accumulation of Na^+^ and proline, and stimulating the expression of stress responsive genes in transgenic plants [45,46,47,48,49,50,51]. Contrastingly, overexpression of *Glycine soja GsTIP2;1* and wheat *TaTIP2;2* decreased drought and salt stress tolerances with relatively low proline content and high water loss in transgenic plants [52,53]. In *Arabidopsis*, microarray analyses showed that most *AtTIPs* were involved in drought, salt, osmotic, ABA, MeJA, GA3, and indole-3-acetic acid (IAA) stress responses (Figure S2). AtTIP1;3 transported water and contributed to plant reproduction. In soybean, *GmTIPs* were uplifted, lowered, or unchanged under drought, heat, flooding, ABA, MeJA, ACC, or SA stress conditions. *GmTIP1;5* responded to drought stress and facilitated the water transport in *Xenopus laevis* oocytes [54]. *GmTIP2;3* improved osmotic stress tolerance in yeast cells [31]. In rice and maize, drought, salt, osmotic, cold, ABA, and GA stresses activated or inhibited the expressions of *OsTIPs* and *ZmTIPs*. *OsTIP1;2* was transcriptionally up-regulated, whereas *ZmTIP1;2* was down-regulated after drought treatment [21,22]. It was urgent to define the precise roles of other *TIP* genes. In the present study, GmTIP1;6 had the highest similarity to TIP1-type proteins from other plants, and the best orthologic match of GmTIP1;6 was GmTIP1;5, AtTIP1;3, OsTIP1;2, and ZmTIP1;2 (Figure 1). The transcripts of *GmTIP1;6* were favorably accumulated after 20% PEG6000, 250 mM NaCl, 100 µM ABA, and 100 µM MeJA treatments in vegetable soybean (Figure 2). *GmTIP1;6* acted as one potential target gene in developing a stress resistant vegetable soybean. The mechanistic pathways behind the distinct roles of *TIPs* remain unelucidated.

Promoter sequences are crucial for the accurate regulation of gene transcriptions in plants. Time, location, and level of gene transcripts affected the functioning of *TIPs* under both favorable and stressful conditions. Many reports have specifically pointed out the compositions of stress and hormone related elements in *TIP* promoters [55,56,57]. Plant responses to stress stimulus were generally mediated by diverse hormonal cues. In rices, the *OsTIP3;1* promoter was reciprocally controlled by ABA and sugar signals through the ACGT and CE1 elements, based on the luciferase (LUC) activity detection in protoplast transient expression assay [58]. In bananas, drought (MBS), cold (LTR), ABA (ABRE), MeJA (CGTCA motif), SA (TCA element), and GA (P-box) responsive elements were observed in the promoter region of *MaTIP1;2*. In soybeans, many light responsive elements, such as Box 4, G-Box and I-Box, GATA, MBS, and GARE motifs were found in the *GmTIP2;3* promoter. Heat (HSE) and ethylene (ERE) responsive elements were detected in the *GmTIP2;6* promoter. GUS activity assays in transgenic plants confirmed that *MaTIP1;2*, *GmTIP2;3* and *GmTIP2;6* promoters were drought, salt, dark, heat, ABA, and ACC stress inducible or inhibitive promoters [19,30,31]. In the present study, the promoter of *GmTIP1;6* was obtained from vegetable soybean. *Cis*-acting elements analyses revealed that *GmTIP1;6* promoter possessed drought (MBS), ABA (ABRE), MeJA (CGTCA and TGACG motifs), GA (P and TATC boxes), and auxin (TGA element) responsive elements (Table 1; Figure 3), which were necessary for the regulation of gene expression. In transgenic *Arabidopsis*, the *GmTIP1;6* promoter directed obvious expression in the vascular tissues of leaves, stems, and roots (Figure 4). Consistent with the expression patterns, the activities of the *GmTIP1;6* promoter were enhanced by drought, NaCl, ABA, and MeJA stresses and varied with different concentrations of stress treatments (Figure 5 and Figure 6). Current data concluded that the *GmTIP1;6* promoter responded to diverse abiotic stresses and hormone signals and regulated gene expression both quantitatively and qualitatively in plants. More experimental evidence is required to establish the detailed regulation mechanisms of the *TIP* stress responsive promoters that are valuable for plant genetic engineering.

## 4. Materials and Methods

### 4.1. Stress and Hormonal Treatments for Vegetable Soybean

The main cultivar of vegetable soybean Zhenong 6 was used in this study. Seedlings were grown in a temperature-controlled chamber (PTC-300, Shanghai, China), and kept at 22 °C day/20 °C night, 16 h photoperiod, 60% relative humidity, and 25,000 Lux light intensity [1]. The root systems of 35 day old seedlings were removed from the soil and immersed into 20% PEG6000, 250 mM NaCl, 100 µM ABA, 100 µM MeJA, 100 nM GA, and 100 nM NAA solutions for 0 h, 0.5 h, 1.5 h, 6 h, 12 h, or 24 h. The seedlings were sampled and frozen in liquid nitrogen and stored at −80 °C for DNA and RNA extractions.

### 4.2. Phylogenetic Analysis for GmTIP1;6

Protein sequences of soybean GmTIPs, *Arabidopsis* AtTIPs, rice OsTIPs, and maize ZmTIPs were obtained as previously reported [19,20,21,22]. Multiple sequence alignment was performed by ClustalX2 software. Phylogenetic tree was constructed by MEGA7.0 software based on the neighbor-joining (NJ) approach followed by 1000 bootstrap replicates [59].

### 4.3. RNA Extraction and qRT-PCR Analysis for GmTIP1;6

RNA was extracted from the vegetable soybean seedlings using the RNAprep Pure Plant Kit (Tiangen, Beijing, China). Then, 1 µg RNA was used for cDNA synthesis with FastQuant RT Kit (Tiangen, Beijing, China). Specific primer pairs for qRT-PCR were designed using PrimerQuest Tool [60] (Table S1). *GmActin11* (Glyma.18G290800) was used as the internal reference [61,62,63]. The amplification reactions of qRT-PCR were performed on Applied Biosystems StepOnePlusTM Real-Time System using SuperReal PreMix Plus SuperReal (SYBR Green) (Tiangen, Beijing, China). The thermal cycle used to the following procedure: 95 °C for 15 min, followed by 40 cycles of 95 °C for 10 s, 53 °C for 20 s, and 72 °C for 30 s, following a 10 min extension at 72° C. Each experiment was accomplished with three technical replicates. The 2^−ΔΔCT^ method was used to analyze the relative amounts of transcripts accumulated for *GmTIP1.6* [64]. Student’s *t*-test was applied to determine significant differences at a level of *p <* 0.01.

### 4.4. DNA Extraction and Promoter Cloning of GmTIP1;6

DNA was extracted from the vegetable soybean seedlings using the Plant Genomic DNA Kit (Tiangen, Beijing, China). Promoter (5′ flanking region upstream of the coding sequence) of *GmTIP1;6* was isolated from the vegetable soybean genomic DNA by PCR. Specific primer pairs were designed for cloning of the *GmTIP1;6* promoter using DNAMAN software (Table S1). PCR product was cloned into pMD-18-T vector (TaKaRa, Shiga, Japan) and verified by sequencing.

### 4.5. Cis-Acting Element Analysis of GmTIP1;6 Promoter

Sequence, number, location, and function of abiotic stress and hormone responsive elements in the promoter of *GmTIP1.6* were analyzed by the PlantCARE database [65].

### 4.6. Vector Construction of GmTIP1;6 Promoter and Transgenic Arabidopsis Generation

To generate the proGmTIP1;6::GUS construct, the promoter of *GmTIP1;6* was inserted into *Pst* I/*Sma* I restriction sites and ligated into the pCAMBIA1391z vector using the primers as presented in Figure S3 and Table S1. The GUS fusion construct was then transformed into *Arabidopsis* by *Agrobacterium*-mediated floral dipping method [66]. Transformed lines were selected using Murashige and Skoog (MS) medium containing 50 mg/L hygromycin (Hyg). The homozygous T3 transgenic *Arabidopsis* seeds were used for further experiments.

### 4.7. Stress and Hormone Treatments for Transgenic Arabidopsis

The proGmTIP2;6::GUS transgenic seeds were germinated on MS medium and cultured in the temperature-controlled chamber (PTC-300, Shanghai, China) kept at 22 °C day/20 °C night, 16 h photoperiod, 60% relative humidity, and 25,000 Lux light intensity for 5 days. Then, the 5-day-old seedlings were exposed to MS medium containing 2–6% PEG6000, 50–150 µM NaCl, 25–50 µM ABA, or 50–100 µM MeJA for 5 days. Seedlings on MS medium without any treatments were used as controls. Each treatment was performed in triplicate. After stress and hormone treatments, the transgenic *Arabidopsis* seedlings were subjected to evaluate the GUS activities.

### 4.8. GUS Staining and Activity Detection for Transgenic Arabidopsis

GUS staining and activity detection were conducted as described previously [19,30,31]. The seedlings of transgenic *Arabidopsis* after staining were photographed using the Leica microscope. GUS activities were analyzed and compared based on Student’s *t*-test, at a significant level of *p <* 0.01.

## Figures and Tables

**Table 1:** Sequence, number, location, and function of *cis*-acting elements in *GmTIP1;6* promoter.

Element Name	Core Sequence	Number	Location (bp)		Function
			(+) Sense Strand	(−) Antisense Strand	
MBS	CAACTG	1	+266		Drought responsive
ABRE-element	ACGTG	5	+1233	−585 −1235	ABA responsive
			+1236		
			+1291		
CGTCA-motif	CGTCA	2	+447	−444	MeJA responsive
TGACG-motif	TGACG	2	+444	−447	MeJA responsive
TCA-element	TCAGAAGAGG	1	+853		SA responsive
P-box	CCTTTTG	1		−1153	GA responsive
TATC-box	TATCCCA	1	+614		GA responsive
TGA-element	AACGA	1	+1413		Auxin responsive

## Data Availability

Data are contained within the article or Supplementary Materials.

## Supplementary Materials

The following supporting information can be downloaded at: https://www.mdpi.com/article/10.3390/ijms232012684/s1.

## Abbreviations

ABA: abscisic acid; ACC, l-aminocyclopropane-l-carboxylic acid; AQP, aquaporin; GA, gibberellin; GUS, beta-glucuronidase; IAA, Indole-3-acetic acid; LUC, luciferase; MeJA, methyl jasmonate; NAA, naphthaleneacetic acid; PEG 6000, polyethylene glycol 6000; qRT-PCR, quantitative real-time PCR; SA, salicylicacid; TIPs, tonoplast intrinsic proteins.
