# Supplementary material for: Promoter of Vegetable Soybean GmTIP1;6 Responds to Diverse Abiotic Stresses and Hormone Signals in Transgenic Arabidopsis
Source: Int J Mol Sci. 2022 Oct 21;23(20):12684. doi: 10.3390/ijms232012684 (PMC9604487; doi:10.3390/ijms232012684)
Supplement: Supplementary file 1 [file ijms-23-12684-s001.zip › ijms-1945009-Supplementary figures.pdf]

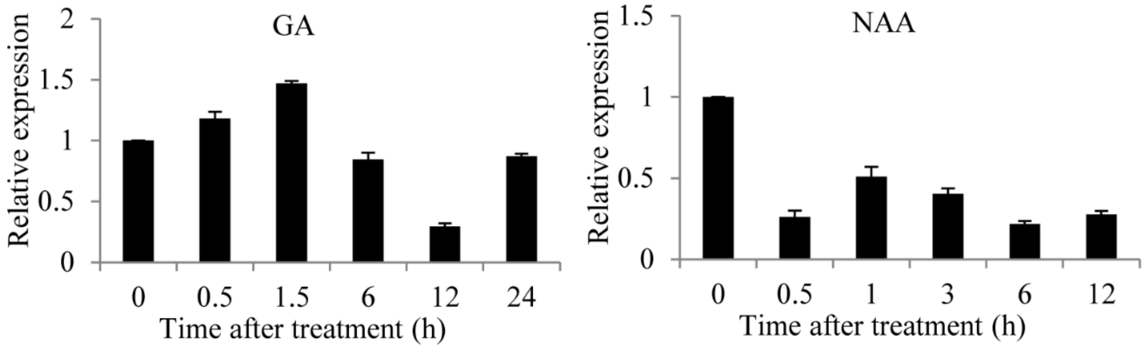

Figure S1. Expression patterns of *GmTIP1;6* under 100 nM GA and 100 nM NAA treatments in vegetable soybean seedlings.

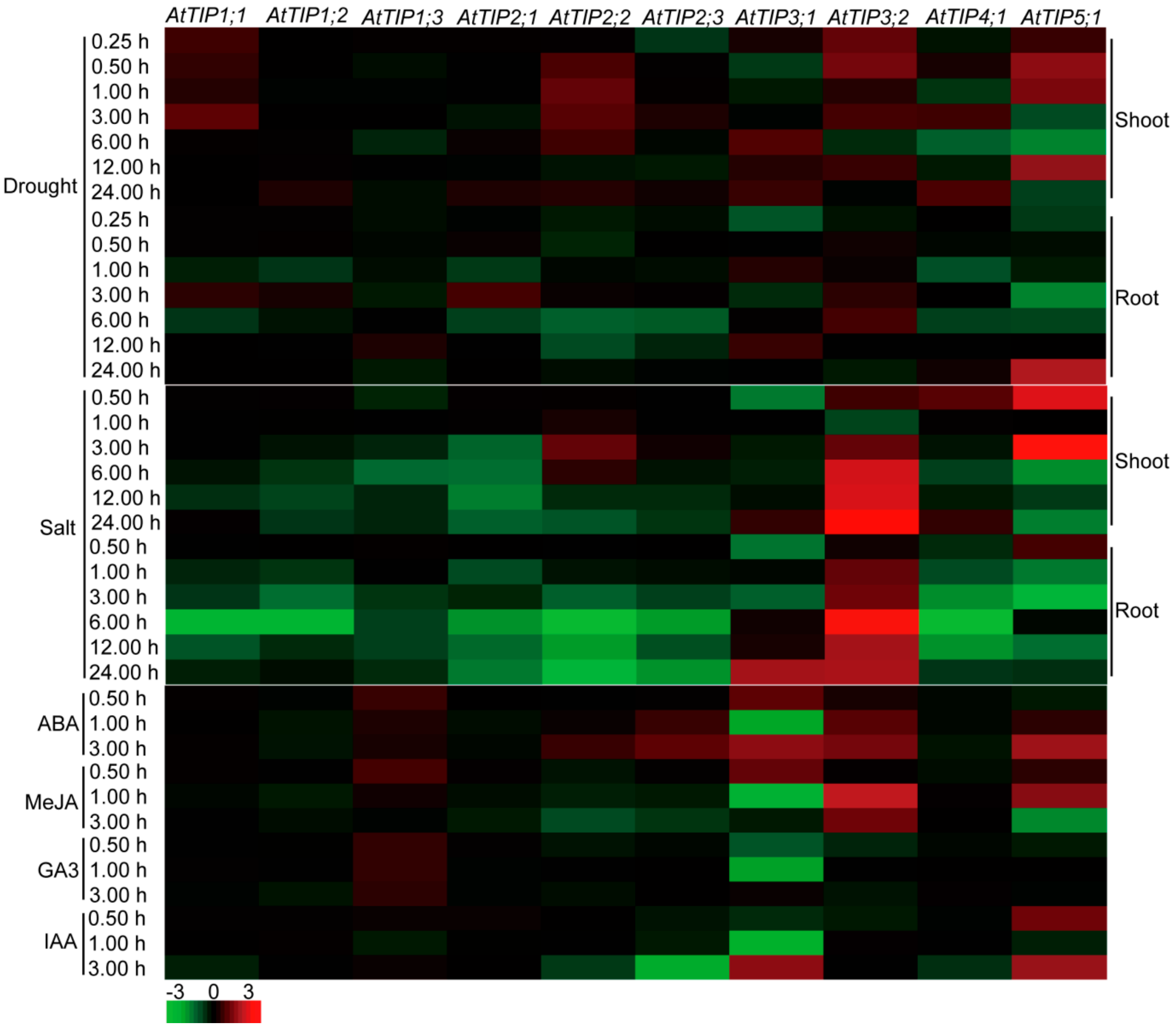

Figure S2. Expression profiles of *Arabidopsis AtTIP* genes in response to drought, salt, ABA, MeJA, GA3 and IAA stresses across seven time points (0.25 h, 0.5 h, 1 h, 3 h, 6 h, 12 h and 24 h) in two tissues (roots and shoots). Differences in gene expression levels were shown in color bars, red: higher expression, green: lower expression.

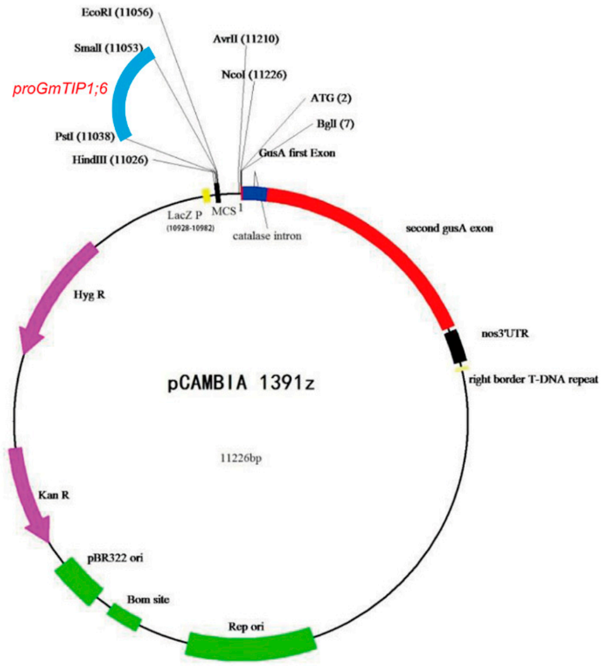

Figure S3. Vector construction of proGmTIP1;6::GUS. Restriction enzyme sites (*Pst* I and *Sma* I) of pCAMBIA1391z were selected for the insertion of promoter.
